# Two-dimensional fruit quality factors and soil nutrients reveals more favorable topographic plantation of Xinjiang jujubes in China

**DOI:** 10.1371/journal.pone.0222567

**Published:** 2019-10-18

**Authors:** Cheng Wang, Weizhong He, Lu Kang, Song Yu, Aibo Wu, Wenliang Wu

**Affiliations:** 1 Beijing Key Laboratory of Biodiversity and Organic Agriculture, College of Resources and Environmental Sciences, China Agricultural University, Beijing, China; 2 Institute of Quality Standards & Testing Technology for Agro-Products, Key Laboratory of Agro-Product Quality and Safety of Xinjiang, Laboratory of Quality and Safety Risk Assessment for Agro-Products (Urumqi), Ministry of Agriculture, Xinjiang Academy of Agricultural Sciences, Urumqi, Xinjiang Province, China; 3 CAS Key Laboratory of Nutrition, Metabolism and Food Safety, Shanghai Institute of Nutrition and Health, Shanghai Institutes for Biological Sciences, University of Chinese Academy of Sciences, Chinese Academy of Sciences, Shanghai, China; Fred Hutchinson Cancer Research Center, UNITED STATES

## Abstract

Jujubes (*Ziziphus jujuba* Mill.) are among the main agroeconomically important crops in Xinjiang, China, and those from this region have the highest production worldwide. However, the reason for the high quality of the jujubes in the region is unknown. In our current research, the total phosphorus (P), total nitrogen (N), organic matter (OM), available P, alkaline N and quick potassium (K) were quantitatively analyzed in soils collected from orchards in 11 geographical locations, counties or cities, in Xinjiang. Meanwhile, the P, total triterpenoids, soluble solids, polysaccharide and cyclic adenosine monophosphate (CAMP) contents were also used to indicate fruit quality. Based on the analyzed data, principal component analysis (PCA) and multiple regression analysis revealed a high correlation between soil nutrients and the quality of the Jun jujube, which was used as an example. Specifically, the total P and quick K contents significantly differed among the orchard soils. Moreover, they significantly affected fruit quality. Total P significantly affected the soluble solids and total triterpenoids contents and was negatively correlated with the former and positively correlated with the latter. In addition, the soluble solids and total triterpenoids contents were significantly affected by the quick K content; as the quick K content increased, the soluble solids content gradually increased, while the total triterpenoids content decreased. According to the response surface model, we suggest that when the total P and quick K contents in the soil in Xinjiang were 0.76 g/kg and 365.04 mg/kg, respectively, the optimal fruit quality was obtained. Therefore, two-dimensional analysis of fruit quality and soil nutrients showed that it is necessary to increase the total P and quick K contents in the soil used to grow jujubes in Xinjiang.

## Introduction

Jujubes (*Ziziphus jujuba* Mill.) are popular because of their attractive taste and texture, and they contain many essential nutrients [1–3]. The main growing regions are distributed in the subtropical and tropical regions of Asia and the Americas [4]. In China, jujubes are mainly distributed in the north. Their fruits are common traditional Chinese medicine and food that have been used for thousands of years [5]. Significantly, 30% of all jujubes worldwide are produced in Xinjiang, at more than 7 million tons per year, ranking China first in production [6]. The quality of Xinjiang jujubes is superior to that of jujubes from other regions because of the area’s unique climatic characteristics, i.e., low rainfall with periodic drought, abundant sunshine, and substantial differences between day and night temperatures [7]. Jujubes are among the main economic crops in Xinjiang. Therefore, it is of great significance to explore their quality factors to improve jujube yield and quality in Xinjiang.

Climatic conditions and soil nutrients are the key factors affecting fruit quality. In the main jujube planting areas in Xinjiang, climatic conditions have been stable. Under stable climatic conditions, soil nutrients are the key factors affecting fruit quality. Several studies have demonstrated that the nutrients in soil, particularly phosphorus (P) and potassium (K), have a significant influence on fruit yield and quality [8–10]. Low P and K contents in the soil can reduce apple production in the Loess Plateau apple producing regions in China [11]. An imbalance in the nitrogen (N)-P-K ratio in soil can adversely affect pear quality [12]. Therefore, ameliorating soil nutrients is among the common methods used to improve fruit quality. In controlled-release fertilizer treatments, the tomato leaf area index at pinching increased linearly with increasing nutrient supply during all cropping seasons [13]. Soil boron (B) fertilization improved root development and tree vigor. The fruits of apple trees fertilized with B were larger and richer in B and had more color and a higher soluble solids content and titratable acidity compared to those of the control trees [14]. When 50 mg/L sodium selenite fertilizer was applied, the vitamin C, soluble sugar and total flavonoid contents of winter jujube increased by 20.94%, 29.48% and 43.48%, respectively [15]. Furthermore, biogas slurry residues contain considerable amounts of plant nutrients. The leaf area index, root length density and grain yield of wheat were higher in plots amended with biogas slurry than in unamended plots [16]. However, very few previous reports have focused on the correlation between soil nutrients and quality in jujubes; most have focused mainly on the evaluation of soil nutrient status and the correlation between single factors of soil nutrients on fruit quality. Thus, the present study sought to analyze the multivariate correlation between the quality of jujubes and soil nutrients in a jujube orchard in Xinjiang and explore the soil nutrient scheme for obtaining high jujube quality in Xinjiang; Jun jujube was taken as an example.

## Materials and methods

### Sample collection

Fifty-five samples of Jun jujube and 55 soil samples from commercial orchards (with the owner's permission) were collected from 11 counties or cities in Xinjiang, China (lat. 36°51′N~41°37′N, long. 77°06′E~82°53′E, elevation, 967.2 m ~ 1388.78 m), during October 2016 (Table 1). Jun jujube samples were randomly collected from selected jujube trees with good growth and without pests and diseases. From each sample, 3 kg of fresh fruit was collected at the end of the ripening stage and then stored at -20°C after crushing. Soil samples were collected using a stainless-steel auger at depths ranging from 0–40 cm; the depth of each position was the same. One representative sample for analysis contained three random samples from a jujube orchard. The soil samples were dried in the shade at room temperature, and then roots and debris were removed. Then, the sample was ground and passed through a 2-mm sieve for analysis.

**Table 1:** Geographical information on the sample orchards.

	Longitude	Latitude	Height (m)
Bachu	39°11′57″	77°39′06″	1165.00
Kuqa	41°36′46.83″	82°50′48.18″	1021.20
Xayar	41°21′44.43″	82°37′22.67″	986.20
Makit	38°58′41.4″	77°38′18.8″	1149.00
Zepu	38°06′30.9″	77°05′41.8″	1336.00
Jiashi	39°41′26.7″	77°13′43.9″	1172.00
Aksu	40°58′02.89″	80°07′00.95″	1071.40
Awat	40°39′45.75″	80°21′28.30″	1053.80
Yutian	36°49′59.9196″	81°33′17.2656″	1388.78
Kunyu	37°16′2.6328″	79°18′55.8144″	1265.33
Qira	36°58′24.0888″	80°48′9.702″	1372.40

### Chemicals and reagents

Glucose, rutin, gallic acid, cyclic adenosine monophosphate (CAMP) and ursolic acid were HPLC grade and purchased from the National Standard Substance Center (Beijing, China). Methanol and potassium dihydrogen phosphate were chromatographic pure reagents purchased from Xi’an Chemical Factory (Xi’an, China). Sodium nitrite, aluminum nitrate, sodium hydroxide, ethanol, methanol, phenol, concentrated sulfuric acid, glacial acetic acid, and vanillin were analytical grade from Beijing Chemical Factory (Beijing, China).

### Quantitative analysis

Fruit quality factors such as polysaccharide, soluble solids and P contents in jujube were detected using Chinese national standard methods GB22221-2008, GB/T 12295–1990 and GB/T5009.87–2003, respectively. The total triterpenoids content was estimated according to a previously published method [17]. The CAMP content was measured based on a previously reported method [18]. The available P, total N, total P, quick K, and organic matter (OM) contents in the soil were measured using Chinese national standard methods NY/T1121.7–2014, GB/T7173-87, LY/T1232-2015, NY/T889-2004 and NY/T1121.6–2006, respectively. Soil alkaline N was ascertained via diffusion plate titration [19].

### Statistical analysis

The experiment was performed using a completely randomized design, and the values obtained for each variable were considered independent for three replications. All statistical analyses, including principal component analysis (PCA) and correlation, were conducted using SPSS V22.0 (IBM, Chicago, USA). The response value of the multiple regression equation was analyzed by design-expert V8.0 (Stat-Ease, Minneapolis, USA).

## Results

### Soil nutrients and fruit quality variables

Six fruit quality variables of Jun jujube and six soil nutrients in orchards from 11 counties or cities were detected in our study. The results indicated that the nutrient contents greatly varied among the different orchard soils. Jiashi county had the highest OM and total N contents, at 13.09 g/kg and 0.88 g/kg, respectively. In addition, the quick K and available P contents were the highest in Yutian county, at 304.29 mg/kg and 41.5 mg/kg, respectively,. In Kuqa and Makit counties, the highest total P content was 0.82 g/kg. Meanwhile, the alkaline N content in KunYu city peaked at 118.07 mg/kg (Table 2). In terms of fruit quality, Jun jujube samples from Yutian county had high soluble solids (61.13%), polysaccharide (27.12 mg/g) and CAMP (702.64 μg /g) contents. The P content (1.45 mg/g) was the highest in the Jun jujubes of Awat county. Kuqa county had the highest total triterpenoids content (20.24 mg/g) (Table 3). The good soil conditions and high-quality fruit in Yutian county indicated that there might be a remarkable correlation between soil nutrients and fruit quality.

**Table 2:** Average soil nutrient contents among the different orchards.

	Total P (g/kg)	Total N (g/kg)	OM (g/kg)	Available P (mg/kg)	Alkaline N (mg/kg)	Quick K (mg/kg)
Bachu	0.68	0.86	11.06	16.84	61.86	182.50
Kuqa	0.82	0.72	8.60	36.16	77.53	232.83
Xayar	0.60	0.57	7.50	13.17	66.92	167.72
Makit	0.82	0.76	12.00	42.28	87.51	250.14
Zepu	0.81	0.62	10.81	30.89	78.21	185.75
Jiashi	0.79	0.88	13.09	39.34	85.97	157.59
Aksu	0.62	0.62	9.41	19.33	65.79	153.33
Awat	0.65	0.51	9.08	18.20	53.84	174.91
Yutian	0.64	0.36	2.64	41.50	74.98	304.29
Kunyu	0.57	0.16	2.44	15.82	118.07	273.27
Qira	0.68	0.49	5.71	15.82	42.36	124.33

**Table 3:** Average fruit quality factors contents among the different orchards.

	P (mg/g)	Total triterpenoids (mg/g)	Soluble solids (%)	Polysaccharide (mg/g)	CAMP (μg/g)
Bachu	1.13	15.38	51.69	221.84	557.93
Kuqa	1.21	20.24	35.94	152.58	472.92
Xayar	1.26	18.63	35.94	78.00	534.20
Makit	1.25	15.08	46.75	140.57	690.94
Zepu	1.15	19.93	47.23	255.80	610.09
Jiashi	1.24	14.23	51.89	227.30	603.61
Aksu	1.22	14.63	38.69	166.84	662.00
Awat	1.45	17.60	42.58	146.35	591.62
Yutian	1.37	10.27	61.13	271.18	702.64
Kunyu	1.19	9.90	55.29	234.79	590.91
Qira	1.23	14.9	50.72	280.36	607.27

### Differential analysis of soil nutrients in different orchards

The main patterns of covariation in the parameters used to describe the soil nutrient and fruit quality variables were evaluated via PCA. This is an exploratory multivariate statistical method that reduces many parameters to a small number of newly derived parameters [20]. Principal components contain the same information as the original parameters but have the advantage of being mutually uncorrelated such that there is no redundant information between them. The results demonstrated representative factors with a significant difference in soil nutrients in different orchards through PCA. The contribution rates of the first and second principal components were 44.066% and 28.206%, respectively, while the cumulative contribution rates reached 72.271%. The characteristic roots were all much greater than 1. The findings indicated that most of the variation in the soil nutrients among the different orchards could be explained by the first and second principal components (Table 4). The largest load in the first and second principal components was that of total P and quick K, respectively, which suggested that these nutrients were important variables for the principal components and could be used as representative factors reflecting differences among soil nutrients between the different orchards (Table 5).

**Table 4:** Principal component characteristic root and contribution rate.

Principal component	Characteristic root	Contribution rate (%)	Cumulative contribution rate (%)
1	2.644	44.066	44.066
2	1.692	28.206	72.271
3	0.802	13.368	85.639
4	0.363	6.053	91.692
5	0.291	4.843	96.534
6	0.208	3.466	100

**Table 5:** Load index of principal component.

	Principal component 1	Principal component 2
OM	0.296	-0.281
Total P	0.326	0.018
Available P	0.262	0.250
Alkaline N	0.164	0.305
Total N	0.285	-0.293
Quick K	0.087	0.520

### Correlation analysis of soil nutrients and fruit quality factors

**Fig 1:**
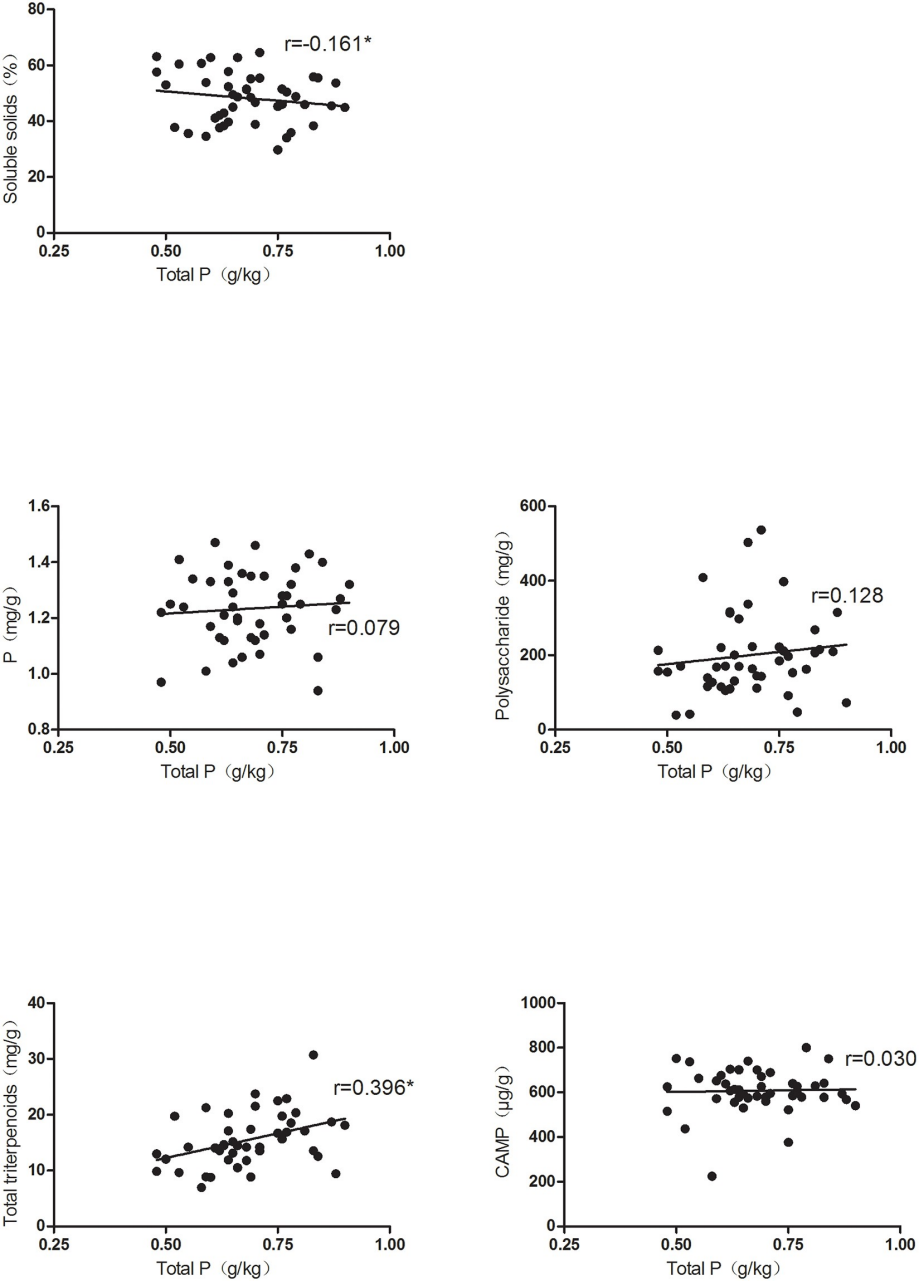
Correlation between total P content and fruit quality variables. The asterisks * indicate significant differences at p< 0.05.

**Fig 2:**
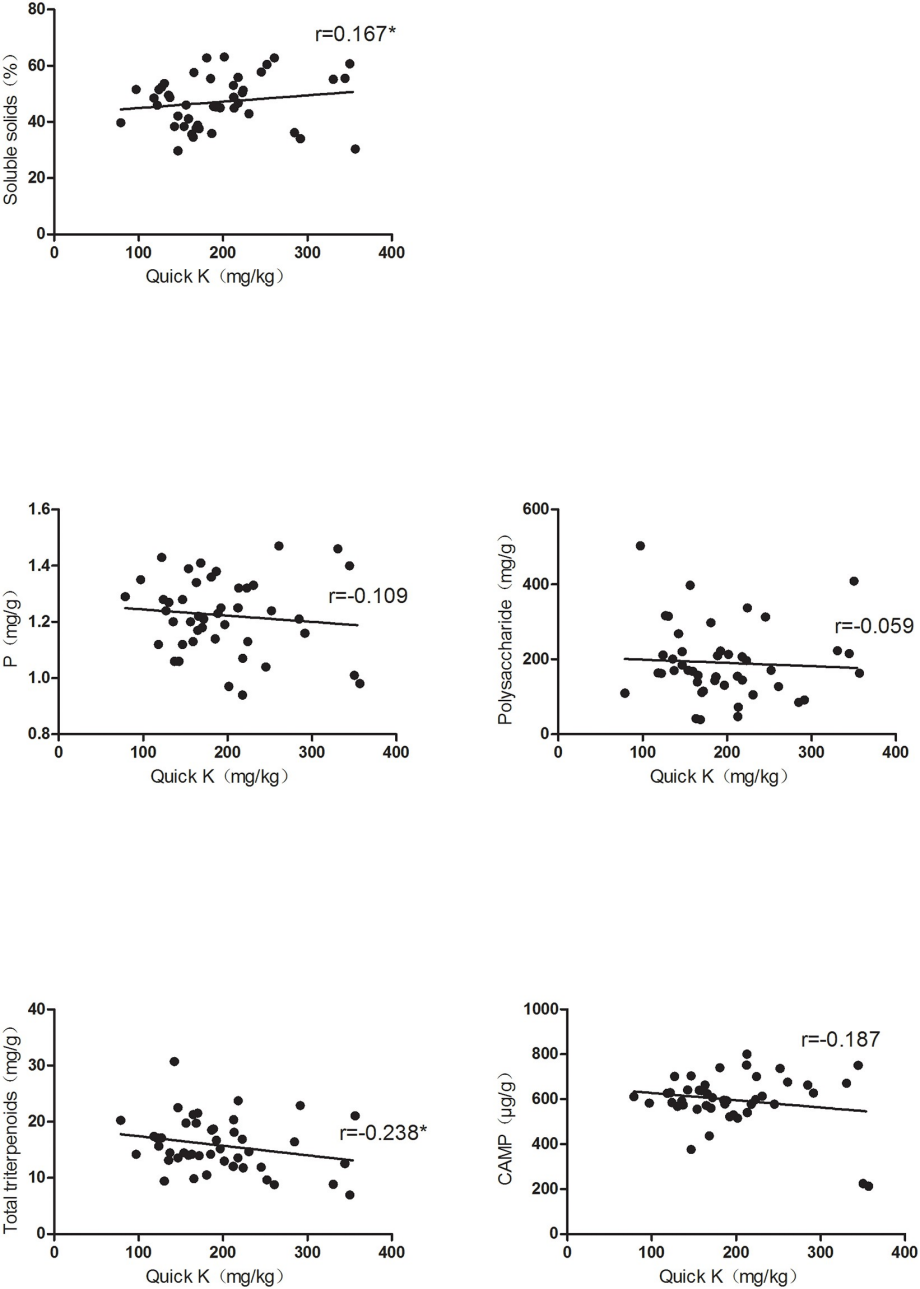
Correlation between the quick K content and fruit quality variables. The asterisks * indicate significant differences at p< 0.05.

Several studies have demonstrated that the fruit quality and yield and soil nutrient contents in orchards are closely related [21, 22]. The correlation between representative soil nutrients and fruit quality factors was analyzed. The results showed that the fruit soluble solids and total triterpenoids contents were significantly impacted by the soil total P and quick K contents. The soluble solids content markedly decreased with an increase in the total P content, while the total triterpenoids content increased (Fig 1). Furthermore, when the quick K content increased, the total triterpenoids content remarkably decreased, while the soluble solids content significantly increased (Fig 2). However, no significant correlation of the total P or quick K content with the other factors was observed in the study. These results implied that different soil nutrients had different effects on fruit quality factors. Therefore, multiple statistical analysis methods were adopted to further explore the relationship.

### Optimization of soil nutrients for high fruit quality

**Fig 3:**
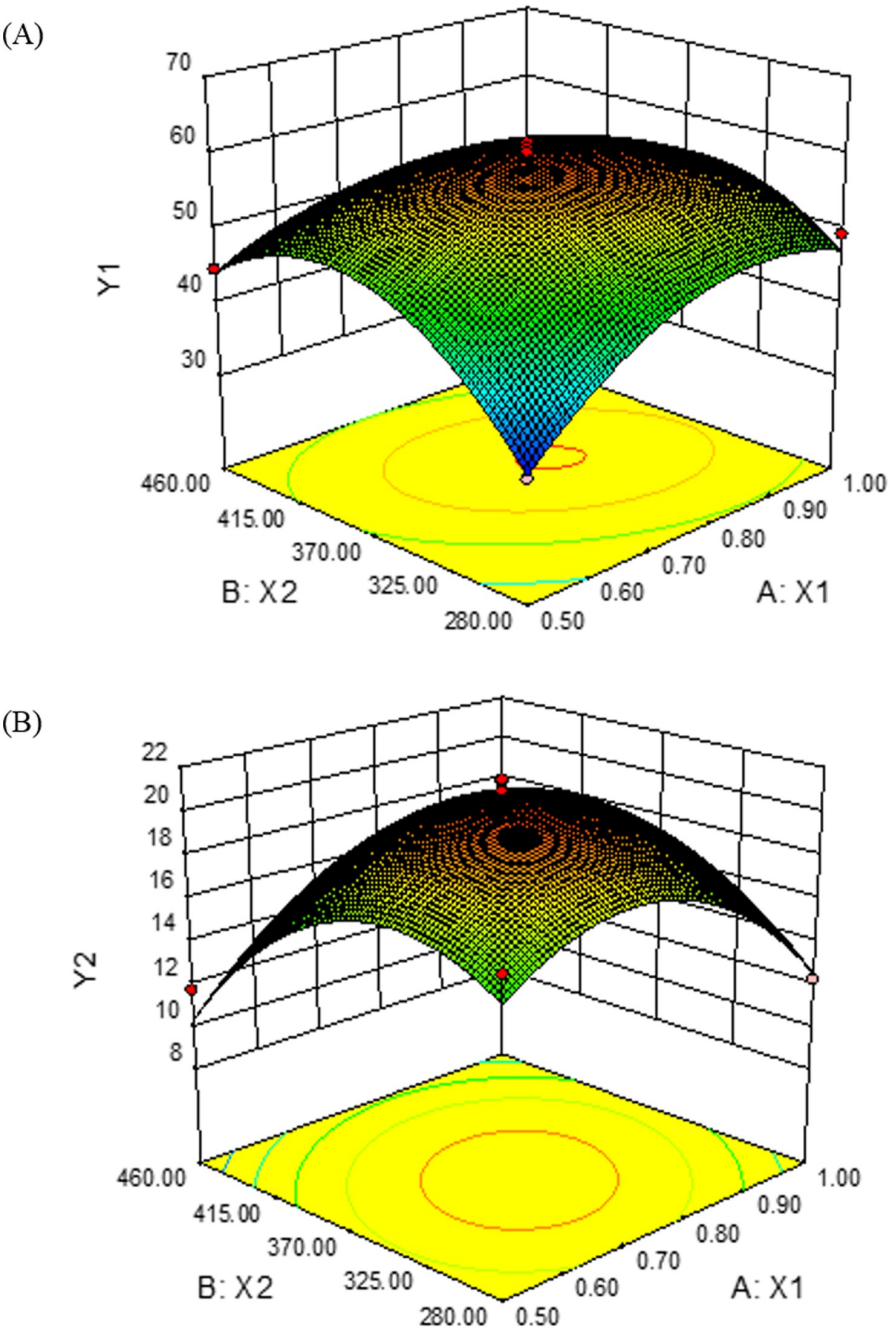
Response surface of the multiple regression equation. (A) Effect of the total P (X1) and quick K (X2) contents on the soluble solids content (Y1). (B) Effect of the total P (X1) and quick K (X2) contents on the total triterpenoids content (Y2).

Considering the previously described total P and quick K content responses, the range of soil nutrient contents in the orchards that obtained high-quality Jun jujubes was explored. The objective of optimizing the soluble solids (Y1) and total triterpenoids (Y2) contents of Jun jujube was used to analyze the suitable range of soil total P (X1) and quick K (X2) contents. Response surface analysis showed that the fitted models explained most of the fruit quality variability in terms of linear, quadratic, and cross product effects of total P and quick K contents. The fitted response surface models were as follows:

$$Y_{1}=-263.75+265.77X_{1}+1.179X_{2}-0.2X_{1}X_{2}-121.36X_{1}^{2}-0.00137X_{2}^{2}$$

(Fig 3A) and

$$Y_{1}=-56.14+65.46X_{1}+0.3X_{2}+0.07X_{1}X_{2}-61.39X_{1}^{2}-0.000496X_{2}^{2}$$

(Fig 3B). These models estimated that the optimal fruit quality was obtained when the total P and quick K contents in the soil were 0.76 g/kg and 365.04 mg/kg, respectively. Under these optimal soil conditions, fruit quality targets of 60.02% soluble solids and 20.88 mg/g total triterpenoids contents can theoretically be achieved. According to our data, the quality of jujubes in most orchards was not optimized, and the total P and quick K contents were insufficient. Therefore, under stable climatic conditions, P and K fertilizer should be reasonably applied to improve the quality of jujubes in Xinjiang.

## Discussion

Xinjiang has stable climatic conditions. In 2016, the average temperature was 12.2°C, the average precipitation was 36.4 mm, and the number of sunshine hours ranged from 2470 to 3000 h. When climatic conditions are stable, soil nutrients become the key factors affecting jujube quality in Xinjiang. In this study, six soil nutrients and five fruit quality variables in samples collected from different orchards were tested. It was found that the soil nutrient contents and Jun jujube quality were significantly different among the different orchards. Yutian county, with fertile soil, had better quality Jun jujubes than those grown in other counties. In our investigation, the correlation between soil nutrients and jujube quality was complicated. Single correlation analysis alone could not fully reflect the influential factors. Therefore, multivariate correlation analysis methods were adopted. Recent studies have reported a correlation between the quality of other fruits and soil nutrient contents in orchards [23–25]. However, limited information is available for the correlation between jujube quality and soil nutrients. In the present study, PCA was used to screen for total P and quick K contents, which significantly differed among the different orchards, and to further explore their effects on jujube quality. The findings indicated that when the total P concentration increased, the soluble solids content markedly decreased, while the total triterpenoids content significantly increased (Fig 1). The quick K content was found to affect the soluble solids and total triterpenoids contents; it was negatively correlated with the latter and positively correlated with the former (Fig 2). These results showed that a fruit quality variable was impacted by different soil nutrients, and this impact might be synergistic or antagonistic. They also showed the complexity of the correlation between soil nutrients and fruit quality.

Soil nutrients are necessary for tree growth and fruit nutrient enrichment. There have been numerous studies of the scientific management of orchard soils as well as reasonable quantitative fertilization formulas [26, 27]. Wang et al. explored the fertilization measures for high yield and a high amylopectin/total starch ratio in the Hongliangfeng 1 waxy sorghum cultivar. They found that the optimal fertilization combination for a yield greater than 5800 kg/ha and an amylopectin/total starch ratio greater than 91% was 262.29–324.30 kg/ha N, 114.07–139.26 kg/ha P2O5, and 230.22–369.28 kg/ha K2O [28]. In our research, multivariate and quantitative statistical analyses were performed to investigate the soil nutrient contents that resulted peak fruit quality. According to our investigation, most of the orchard soil nutrients did not reach the optimal level; thus, increasing the amount of P and K fertilizer applied may improve jujube quality in Xinjiang.

## Conclusions

We found that the nutrient contents greatly varied among the different orchard soils. The total P and quick K contents were significantly different among the soils from the 11 counties or cities. They significantly influenced Jun jujube quality (soluble solids and total triterpenoids contents). Through multiple regression analysis, we established response surface models. The fitted response surface models were as follows:

$$Y_{1}=-263.75+265.77X_{1}+1.179X_{2}-0.2X_{1}X_{2}-121.36X_{1}^{2}-0.00137X_{2}^{2}$$

and

$$Y_{1}=-56.14+65.46X_{1}+0.3X_{2}+0.07X_{1}X_{2}-61.39X_{1}^{2}-0.000496X_{2}^{2}.$$

We predicted that the optimal fertilization combination for fruit qualities of 60.02% soluble solids and 20.88 mg/g total triterpenoids was 0.76 g/kg total P and 365.04 mg/kg quick K. The quality of jujube may be improved by properly using P and K fertilizer in Xinjiang. In the future, field simulation experiments are needed to verify the scheme rationality and determine the optimal fertilizer application range.

## Supporting information

S1 Table Raw data. This table contains original information on the location, soil nutrient contents and fruit quality factors of the samples.
(XLSX)
